# Determination of pH in Regions of the Midguts of Acaridid Mites

**DOI:** 10.1673/031.010.4201

**Published:** 2010-05-08

**Authors:** Tomas Erban, Jan Hubert

**Affiliations:** Crop Research Institute, Drnovska 507, Praha 6, Ruzyne, CZ-16106, Czechia

**Keywords:** acidobasic indicators, digestion, enzyme, gut, lumen conditions

## Abstract

The pH of the guts of mites strongly affects their digestive processes. This study was carried out to determine the pH in the guts of 12 species of stored product and house dust mites. Eighteen pH indicators were chosen and offered to the mites in the feeding biotest. Based on the color changes of the indicators, the gut contents of acaridid mites were determined to be within a pH range of 4 to neutral. The gut contents showed a gradient in pH from the anterior to the posterior part. The anterior midgut (ventriculus and caeca) of most species had a pH ranging from 4.5 to 5, or slightly more alkaline for most of the species, while the middle midgut (intercolon/colon) had a pH of 5 to 6. Finally, the pH of the posterior midgut (postcolon) was between 5.5 and 7. Except for *Dermatophagoides* spp., no remarkable differences in the pH of the gut were observed among the tested species. *Dermatophagoides* spp. had a more acidic anterior midgut (a pH of 4 to 5) and colon (a pH of 5) with postcolon (a pH of below 6). The results characterizing *in vivo* conditions in the mite gut offer useful information to study the activity of mite digestive enzymes including their inhibitors and gut microflora.

## Introduction

Synanthropic mites are important pests, and their gut pH strongly affects acaricidal functions, pathogen infection success, and digestive processes. It is necessary to characterize the gut pH first to study the activity of pest digestive enzymes and gut microbial flora, (Skibbe et al. 1996; Terra et al. 1996; Funke et al. 2008). Although several studies determining pH in the gut have been conducted, correctly determined pH values in the digestive tract of synanthropic mites are lacking.

In general, colorimetry, potentiometry, and conductometry are useful methods for the determination of pH (Carsky 1981). Microelectrodes are the most practical and most frequently used tools to determine the pH in insects (Brune et al. 1995; Harrison 2001; Zimmer and Brune 2005; Gross et al. 2008), but this is not possible in mites due to their small body size. Skibbe et al. (1996) showed nuclear magnetic resonance microscopy as an alternative method for determination of pH in insect guts, but this method is not commonly used.

Colorimetric determinations of pH use halochromic chemical compounds as pH indicators. The pH-dependent color changes of acid-base indicators are described by Ostwald's theory, which was later improved by Hantzsch for tautomery conception: such that the human eye is able to perceive color changes of two diverse colored forms at concentration ratios from 10:1 to 1:10, so that the transition range is about pH 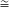 p*K*_A_ (HIn) ± 1, where p*K*_A_ (HIn) is the dissociation constant of the indicator. According to Ostwald's theory, pH indicators are weak acids or bases, and the pH closest to the equivalence point is the titration exponent pT. The color change depends on the presence of dissociated and non-dissociated forms of a given indicator, which results from the activity of hydronium ions (H_3_O^+^). The indicators do not change sharply at a particular pH, but rather change over a narrow range of pH values. Indicators exhibit intermediate colors at pH values inside these transition ranges (Brdicka and Dvorak 1977; Dvorak and Koryta 1983; Holzbecher and Churacek 1987).

Although the results obtained using pH indicators correspond to potentiometric determinations (Espinoza-Fuentes and Terra 1987; Boudko et al. 2001), potentiometry is a more exact method than colorimetry. Due to their size, the only useful method for determining pH in the digestive tract of acaridid mites is colorimetry. The fact that a mite's body is thin and transparent makes it suitable for the application of colorimetry and the direct determination of the gut pH using a compound microscope. This method was previously applied to determine the physiological pH in the gut using several pH indicators such as litmus, phenol red, universal indicator, and neutral red (Hughes 1950; Akimov and Barabanova 1976, 1978; Akimov 1985). The present study is the first report of the pH in the gut of stored product and house dust mites, based on colorimetrical determinations using 18 indicators.

## Materials and Methods

### Experimental mites

Twelwe species of acaridid mites (Acari: Astigmata) from the following families were tested including 5 species of Acaridae (*Acorus siro* Linnaeus, *Aleuroglyphus ovatus* Troupeau, *Sancassania rodionovi* Zachvatkin (syn. *Caloglyphus redickorzevi* Zachvatkin and *Caloglyphus hughesi* Samsinak), *Tyrophagus putrescentiae* Schrank, and *Tyroborus lini* Oudemans); Carpoglyphidae (*Carpoglyphus lactis* Linnaeus); Chortoglyphidae (*Chortoglyphus arcuatus* Troupeau); 2 species of Pyroglyphidae (*Dermatophagoides farinae* Hughes and *Dermatophagoides pteronyssinus* Trouessart); and 3 species of Glycyphagidae (*Glycyphagus domesticus* De Geer, *Lepidoglyphus destructor* Schrank, and *Aëroglyphus robustus* Banks). These species were maintained in laboratory colonies kept in the Crop Research Institute, Prague, Czechia (for a detailed description of rearing conditions, see Erban and Hubert (2008)).

### Application of indicators and preparation of mites for microscopic observation

The selection of indicators was based on their pT and/or p*K*_A_ values and transition ranges (Holzbecher and Churacek 1987, Senese 2000, Shevchenko and Kulichenko 2005, Sabnis 2008). All indicators were purchased from Sigma-Aldrich. The indicators were separated into six groups: (1) Indicators determining the pH between 3 and 8 were Phenolphthalein (p*K*_A_ = 9.1, 9.4, 9.53, 9.70; pT = 8.4–9.4; transition range = 8.3–10.0) and thymol blue (p*K*_A_ = 1.65; 8.90; pT = 2.6 and 8.4; transition range = 1.2–2.8 and 8.0–9.2); (2) Indicators determining pH values near neutral pH were litmus (p*K*_A_ = 7.00; pT = NF (not found); transition range = 5.0–8.0), phenol red (p*K*_A_ = 7.9; pT = 7.0; transition range = 6.8–8.2), brilliant yellow (p*K*_A_ = NF; pT = NF; transition range = 6.5–8.0) and bromothymol blue (p*K*_A_ = 7.0, 7.1; pT = 6.4; transition range = 6.0–7.6); (3) Indicators determining the basic limit of gut pH were bromophenol red (p*K*_A_ = 6.51; pT = 6.16; transition range = 5.2–6.8) and chlorophenol red (p*K*_A_ = 6.00; pT = 6.00; transition range = 4.8–6.4); (4) Indicators determining the lower (acidic) limit of gut pH were bromophenol blue (p*K*_A_ = 4.0, 3.85, 3.6; pT = 4.0; transition range = 3.0–4.6), alizarin red S (p*K*_A_ = 4.5; pT = NF; transition range = 3.5–6.5), methyl orange (p*K*_A_ = 3.76, 3.40; pT = 4.0; transition range = 3.0–4.4) and congo red (p*K*_A_ = 4.1; pT = NF; transition range = 3.0–5.0); (5) Indicators determining pH more accurately within the acid and base limits were methyl red (p*K*_A_ = 5.06, 2.3, 2.5, 4.95; pT = 4.80; transition range = 4.4–6.2), resazurin (p*K*_A_ = 6.71; pT = NF; transition range = 3.8–6.5) and bromocresol green (p*K*_A_ = 4.7, 4.6; pT = 4.66; transition range = 3.8–5.4); (6) Universal acidbase indicators (UABI) were UABI 3–10 (transition range = 3.0–10.0), UABI 0–5 (transition range = 3.0–10.0) and UABI 4–10 (transition range = 4.0–10.0).

The indicators were dissolved in water or a water-ethanol solution and transferred into ground wheat to obtain a 2% final concentration (wt/wt) of indicator. The moistened indicator-enriched diet was mixed using a vortex (IKA® Works, Inc., www.ika.com) and then placed in a sealed glass tube for freeze drying (Heto PowerDry LL3000, Thermo Fisher Scientific, www.thermo.com). The dried indicator-enriched diet was pulverized using a porcelain mortar. About 5 mg of the powder was transferred into 1.5 ml microcentrifuge tubes (Eppendorf, www.eppendorfna.com) and at least 50 specimens were added. A piece of moistened filter paper was added to each tube. In intervals of 24, 48 and 72 hours, the mites were removed and placed in a glass Petri dish. After the mites were scattered, the specimens that had ingested indicator were collected using a camel hair brush under a Stemi 2000-C dissection microscope (Carl Zeiss, www.zeiss.com). The mites were rinsed in a drop of physiological solution (0.9% NaCl) to remove external food particles before being examined by light microscopy.

### Microscopic observations of mite gut

**Figure 1.**
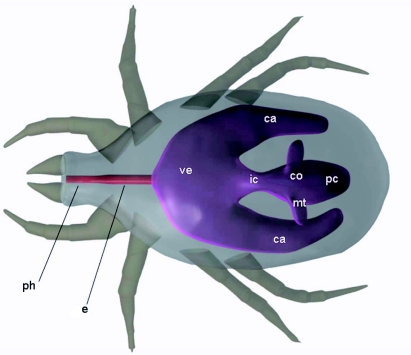
3D model of mite digestive tract. Legend: ca — caeca; co — colon; e — esophagus; ic — intercolon; mt — malphighian tubes; pc — postcolon; ph — pharynx; ve — ventriculus High quality figures area available online.

The color changes of the gut were observed under a compound microscope Axioskop using Axiovison software (Carl Zeiss) and a Powershot A620 digital camera (Canon Inc., www.canon.com). The minimal design included 10 positive observations per species per indicator. Unlike in histological studies (Sobotnik et al. 2008), the intercolon could not be distinguished from the gut during observation. In the present study, the color changes of the indicators in the ventriculus, caeca, colon, and postcolon were determined, and the intercolon was excluded (for detail of gut anatomy, see Figure 1). The color changes of the dissolved dyes in the gut lumen were taken into account, and the color changes of food or fecal boli (which contained highly concentrated indicators) were omitted. To generate color change controls, the color indicators were dissolved in Britton Robinson I buffer. This was made with a precision of 0.1 pH units, and the color changes were compared to the colors in the microscopic observations.

## Results

Mites not feeding on the indicator diet had transparent bodies. The ingestion of indicator diets changed the color of the gut (Table 1).

### Indicators determining the pH between 3 and 8

Phenolphthalein was always colorless and corresponded to observations without any indicator. Thymol blue was always yellow and no exceptions were found.

**Table 1.**
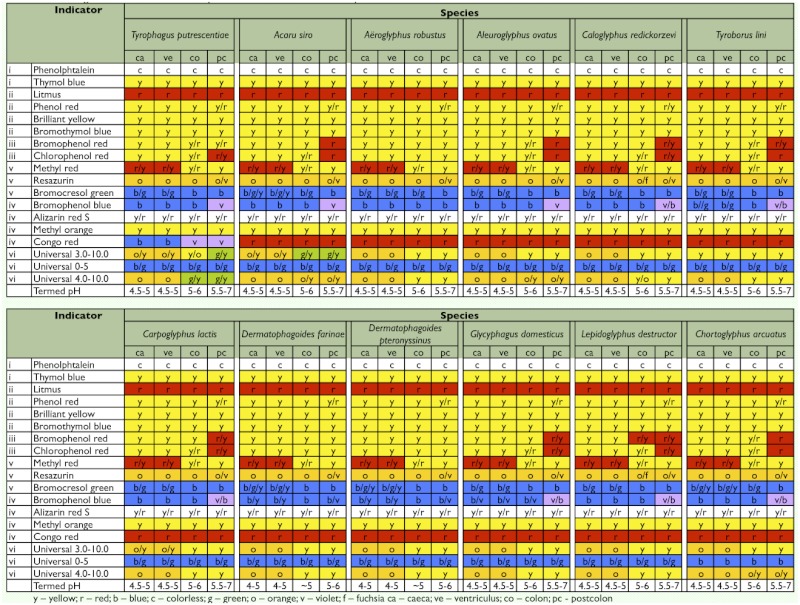
Indicators used in the tests are shown alongside parameters and color changes observed in various parts of the gut of tested mites. The colors of the indicators resulting from the test are denoted by the color of the table as well as by letters.

### Indicators determining pH values near neutral pH

The indicators displayed a yellow color in all cases, except red-colored litmus and a seldom-seen red coloration of the postcolon by phenol red. The color changes indicated that the pH in the gut was generally more acidic than a pH of 6.5. There were observed interspecies differences in the color of phenol red in the postcolon of all species tested. The red-colored postcolon indicated a pH of about 7 in some cases.

### Indicators determining the basic limit of gut pH

This group of indicators showed that the upper limit on the pH scale was about 6.0. The indicators together showed a pH of less than 6 in the caeca and the ventriculus of all tested species of mites. In all species, the colors of both indicators were yellow in the ventriculus and caeca. In the colon and postcolon, a red color was observed, and the intensity of red coloration significantly increased from colon to postcolon. Only *Dermatophagoides* spp. and *A. robustus* showed yellow coloration of the whole gut.

**Figure 2.**
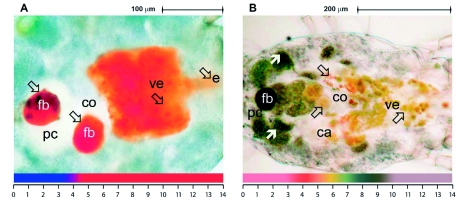
The coloration of the following acid-base indicators in the gut of specimens: A) Congo red (*Dermatophagoides farinae*); B) Universal indicator pH 3–10 (*Tyrophagus putrescentiae*). Transparent arrows show observed colors in the mesodeal compartments, while black arrows indicate colored tissues. Legend: ca — caeca; co — colon; e — esophagus; fb — food bolus; pc — postcolon;, ve - ventriculus. High quality figures are available online. The Appendix shows the response of tissues to indicators in more detail.

### Indicators determining the acidic limit of gut pH

Generally, the color changes of the indicators showed a pH higher than 4 in the mesodeal lumen. Bromophenol blue typically resulted in a blue coloration of the caeca, ventriculus and colon. However, a different situation was found in both *Dermatophagoides* species where they were yellow or green. In one specimen of *T*. *lini*, a green/blue coloration was observed in the ventriculus and caeca. Methyl orange and congo red (Figure 2A) produced similar colors in all mesodeal compartments among all species tested. Methyl red produced a yellow color and congo red (Figure 2A) produced a red color throughout the whole gut. Alizarin red S produced a red, or yellow, colored gut and did not correspond with other indicators.

### Indicators determining pH more accurately within the acid and base limits

Methyl red indicates a pH below 5 when it is red and a pH above 5 when it is yellow. The predominantly red-colored methyl red caeca and ventriculus were therefore indicative of a pH below 5. On the other hand, a predominant yellow color was found in the colon, indicating a pH higher than 5. A yellow postcolon indicated a pH more basic than pH 6. This is in respect to the methyl red transition range (4.4–6.2) and the absence of yellow color below pH 6.2. Methyl red nicely illustrated the pH gradient across the whole gut from the middle acidic caeca and ventriculus to the rather neutral postcolon. Resazurin changed color from orange to violet within these values. Based on the titration, the fuchsia color is recognizable from a pH of about 5 and more alkaline, while violet is visible from a pH of about 5.5 and more alkaline. The prevailing color in the gut was orange. There were rare situations in which specimens had a fuchsia color in the colon and a violet color in the postcolon. Bromocresol green becomes green within the narrow pH range of 4.2 and 4.7, while the cyan blue range is between 4.8 and 5. The yellow color indicates a pH more acidic than 4.2. Between pH values of 5.1 and a neutral or alkaline pH, the indicator is blue. The ventriculus and caeca of most species were colored blue and/or green. An exception was found, however as additional yellow coloration was found in the ventriculus and caeca of four species of mites. While *A. siro* and *C*. *arcuatus* had, in one case, a yellow color mixed with blue, some specimens of *Dermatophagoides* spp. had a mostly yellow foregut. Generally, the blue coloration in the colon and postcolon did not show significant color differences between species. Additional green coloration in the colon in some cases further indicated that the colon is more acidic than the postcolon and more alkaline than the ventriculus and the caeca.

### Universal acid-base indicators

This group of indicators represented cocktails that were able (with some limitations) to indicate pH with an accuracy of 1 pH unit. UABI 3–10 (Figure 2B) showed a light orange color in the ventriculus and caeca, corresponding to a pH between 4 and 5. The light orange color changed to yellow in both the colon and the postcolon, indicating a pH around 6 ± 0.5. The green coloration of the colon or postcolon indicated the possibility of a more alkaline environment. In addition, green coloration indicated a pH of about 7 inside the mesodeal cells and ovaria. Generally, the color produced by UABI 0–5 was blue/cyan or blue in the whole gut, indicating a pH higher than 4 for all compartments. In general, UABI 4–10 turned from orange in the ventriculus and caeca to yellow in the colon and postcolon. The orange color of UABI 4–10 indicated a pH below 5 in the ventriculus and the caeca; the yellow color indicated a pH between 5 and 6.5 in the colon and postcolon, and the green coloration indicated a pH of about 7 inside the mesodeal cells and ovaria, similar to the results from UABI 3–10 (Figure 2B).

## Discussion

The method described here can be evaluated as a universal approach for the determination of pH by acidobasic pH indicators in the arthropod gut, where the only necessary condition is the transparency of the arthropods' bodies or dissection of the gut (Terra and Regel 1995). Acidobasic indicators are suitable for the determination of pH if the size of the sample does not allow for the usage of microelectodes, which offer much more precise measurements. The disadvantages of acidobasic indicators lie mainly in the subjectivity of the determinations, since the pH indicators are susceptible to imprecise readings. For precise determination of pH using indicators, it is necessary to use many of them, since they do not change color sharply at one particular pH. Every pH indicator has a tabulated transition range, but none determine pH exactly. The color inside the transition range goes from a lower (more acidic environment) to an upper (more alkaline environment) pH limit, but the human eye starts to detect the second color at some point near the titration exponent (pT). The pT can help us in evaluation of the data, but it is necessary to note that it has some limitations. These are the main reasons why such a large palette of pH indicators is needed for the determination of pH in the gut compartment.

The use of universal acid base indicators as an initial step in the pH determinations may offer a prescreened pH in the gut, possibly reducing the number of indicators used. In this case, however, it could eliminate only the use of Phenolphthalein and thymol blue. If prescreening is used, there is the possibility of making subjective decisions that could affect the results (Barrow and Conrad 2006). At least several, never one or two, pH indicators must be always used in determination of pH to obtain objective data, because each pH indicator can determine the pH only very approximately with an accuracy of pH ± 1. The physicochemical theories of Ostwald and Hantzsch must be respected in this regard also in evaluation of the data. For example, the approximation of the average value inside the transition range is not accurate.

An initial study of pH in the gut of mites showed that pH in the ventriculus and caeca of *A. siro* (syn. *Tyroglyphus farinae*) was between 5.0 and 6.0, while the pH in the colon was determined to be above 7.0 and below 8.0 (Hughes 1950). More recently, the pH in the gut of 16 mites was determined to be in the range of 5.4 to 6.3 in the caeca and ventriculus, 5.9 to 7.4 in the colon, and 6.8 to 8.0 in the postcolon (Akimov and Barabanova 1976, 1978; Akimov 1985). Because these results were obtained using a limited number of indicators (litmus, phenol red and neutral red), some inaccuracies would be expected. In addition, the determination of pH to an accuracy of 0.1 pH unit is impossible according to the laws of physical chemistry. For example, litmus is a pH indicator that generally determines acid or base pH since it is purple at about a pH of 7. The transition range of litmus is wide and ranges from 4.5 to 8.3. The purple color determined by Hughes in the colon of A. siro could determine pH somewhere inside the transition range, starting at a pH of about 6 to 7 (the red and blue are equal). At more acidic values, this indicator starts to become blue, which was not observed. The purple color of litmus, indicating neutral pH, was recorded in our study only for food boli in the postcolon, where we suggest that the color could be affected by a higher concentration of indicator. The neutral red and phenol red showed a pH more acidic than 7 only in the ventriculus, caeca and colon. Only the universal indicator could determine the pH more accurately, but universal indicators usually have the same color in a range of at least 1 or 2 units.

Nevertheless, based on these previous studies, the basic limit of the pH in the gut was near or below pH 8, while the acidic limit was unclear. The same results can be obtained from the determination of pH using Phenolphthalein and thymol blue. In addition, the thymol blue results exclude strong acidic pH (below pH 3). According to the results of two of the groups of indicators (indicators determining pH values near neutral pH and indicators determining the basic limit of gut pH), the basic pH limit of the postcolon and colon were 6.5–7 in the postcolon and about pH 6 in the colon. The caeca and ventriculus both had a pH lower than 6. *Dermatophagoides* spp. and *A. robustus* had different properties, with a pH of about 6 in both the postcolon and the colon. Indicators determining the acidic limit of gut pH, in general, showed a pH higher than 4 or near 4 throughout the whole gut. The results of Alizarin red S tests led to the conclusion that the presence of yellow and red in all compartments was affected by the extremely wide pH transition range (pH 3.5–6.5). Second, the coloration was affected by the presence of Ca^2+^ ions (Dahl 1952). Both *Dermatophagoides* mites displayed tendencies for buffering at pH 4 in the caeca and ventriculus. The use of indicators determining pH more accurately within the acid and base limits showed a more acidic pH in the gut of both *Dermathopagoides* species. This corresponds to their feeding on protein-rich food sources and the presence of cysteine proteases (formerly allergens Der p1 and Der f1) (Tovey et al. 1981). Secretion and synthesis of Der p1 in the cells of the posterior ventriculus was shown using immunostaining of *D. pteronyssinus* sections (Tovey and Baldo 1990). In insects, the acid pH optima of cysteine proteases are well documented (Terra and Ferreira 1994) and could also be expected in mites.

As shown here, indicators determining pH more accurately within the acid and base limits provide for a more exact determination of pH in the morphological compartments of the mite gut, including (a) the anterior gut (the ventriculus and caeca), with a pH range of 4 to 5; (b) the midgut (intercolon/colon), with a pH between 5 and 6; and (c) the postcolon, with a pH ranging from 5.5 to 7. In addition, using the indicators red/yellow methyl red and blue/green bromocresol green, the pH in caeca and ventriculus of the mites (except *Dermatophagoides*) was determined more precisely in range from 4.5 to 5 or slightly more alkaline. *Dermatophagoides* spp. had a more acidic anterior gut (a pH from 4 to 5), colon (a pH of 5) and postcolon (a pH of below 6).

The pH of the midgut lumen is, in most cases, actively regulated and varies with the phylogeny and feeding ecology (Harrison 2001). The gut physicochemical properties are optimized with respect to utilized food source (Zimmer and Brune 2005). There is a relationship between mesodeal pH and the pH optima of some digestive enzymes that hydrolyze the nutrients in the lumen contents (Terra et al. 1996; Funke 2008). The pH in the gut is important not only for the digestive enzymes, but it also influences the solubility of food components, the dissociation or coagulation of ingested proteins, and the presence of gut microflora (Funke et al. 2008). In mites, the known pH optima of enzymes correspond, in some cases, to the observed pH in the gut. The lysozyme pH optima of 4.0 to 5.0 corresponded to the lumen pH in the ventricles and caeca, where lysozyme activity was localized (Erban and Hubert 2008). A similar situation was observed for α-amylases and α-glucosidases with pH optima from 5 to 6 (Akimov and Barabanova 1976a, 1976b, 1978; Akimov 1985; Sanchez-Monge et al. 1996; Hubert et al. 2005; Hubert et al. 2007). However, the ranges of the pH optima for cellulase (5–7.5) and chitinases (5–8) are not in accord with the pH of the gut lumen in many of the species tested (Akimov and Barabanova 1976a, 1976b, 1978; Akimov 1985). This suggests that these enzymes do not have digestive functions in the gut lumen.

Proteases were extensively studied in acaridid mites due to their allergenic importance (Tovey et al. 1981). The enzymatic analyses were based usually on both specific and non-specific substrates combined with the panels of inhibitors (Ortego et al. 2000, Monteallegre et al. 2002, Sanchez-Ramoz et al. 2004). *In vitro*, the enzymatic activities were measured usually in the alkaline environment. For example, mite proteases measured using azocasein as the nonspecific substrate, showed pH optima at 6.0 in body extracts and 9.5 or 10.0 in feces. The pH optima of proteolysis of haemoglobin at pH 3 or 4 indicated the presence of non-digestive acid proteases. The pH optima of the specific activities of serine and cysteine proteases were determined to be near pH 7 or 10.5 in both mite bodies and feces (Ortego et al. 2000, Sanchez-Ramoz et al. 2004). Monteallegre et al. (2002) measured activity and inhibition of serine proteases of mite bodies at pH 7.8 and 7.4.

In previous studies pH measurements were not determined in the mite gut under physiological conditions. According to the results of the present study, the alkaline gut environment does not correspond to the pH optima of the digestive enzymes. Thus, these proteases are not functional (Terra and Ferreira 1994). The study of digestive enzymes needs to be focused on the physiological gut pH.
